# Three new species and four new records of the genus *Aleuroclava* Singh (Hemiptera, Aleyrodidae) from China, with the first description of seasonal dimorphism in the genus

**DOI:** 10.3897/zookeys.1272.148064

**Published:** 2026-03-05

**Authors:** Qing-Song Lin, Ji-Rui Wang

**Affiliations:** 1 Key Lab for Biology of Crop Pathogens and Insect Pests and Their Ecological Regulation of Zhejiang Province, College of Advanced Agricultural Sciences, Zhejiang Agriculture & Forestry University, Lin’an, Zhejiang 311300, China Zhejiang Agriculture & Forestry University Lin’an China

**Keywords:** Aleyrodinae, morphology, puparia, puparium, taxonomy, whitefly

## Abstract

Three new species of the genus *Aleuroclava* Singh viz., *A. bannanensis***sp. nov**. (Yunnan: Xishuangbanna), *A. rubi***sp. nov**. (Zhejiang: Hangzhou), and *A. yunnanensis***sp. nov**. (Yunnan: Xishuangbanna) are described and illustrated. And four species of the genus, viz. *A. bifurcata* (Yunnan: Xishuangbanna), *A. citrifolii* (Yunnan: Xishuangbanna), *A. pearlis* (Yunnan: Xishuangbanna), and *A. stereospermi* (Yunnan: Pu’er) are new records to the known fauna of China. In addition, the seasonal dimorphism changes of *A. montanus* are recorded for the first time, and comments on its morphology are provided. This brings the number of Chinese species of *Aleuroclava* to 44. Images of the habitus, line illustrations, and scanning electron microscope photomicrographs are provided.

## Introduction

The whitefly genus *Aleuroclava* was erected by Singh (1931) with *Aleuroclava complex* Singh, 1931 as its type species by monotypy. It is by far the largest genus of whiteflies, with 148 nominal species (Josephrajkumar et al. 2024). The majority of species occur in the Oriental and Austro-Oriental regions, but also occur in the Palaearctic and sub-Saharan Africa regions (Dubey and Sundararaj 2005). So far, 37 species are clearly known in China (Martin and Mound 2007; Evans 2008; Wang et al. 2014; Wang and Du 2015; Wang and Du 2016), and this work brings the number of Chinese species of *Aleuroclava* to 44. *Aleuroclava* is a large genus with high diversity and complexity in its dorsal and setae structures. Five genera have been synonymized with *Aleuroclava* viz., *Aleurotuberculatus* Takahashi, 1932, *Hindaleyrodes* Meganathan & David, 1994, *Japaneyrodes* Zahradnik, 1962, *Martiniella* Jesudasan & David, 1990 and *Taiwanaleyrodes* Takahashi, 1932. So far, there is no consensus on whether the characteristic of “having two-jointed tuberculate setae” represents a valid genus. Therefore, Josephrajkumar et al. (2024) recently established the *Aleuroclava canangae* (Corbett, 1935) species group to accommodate these species.

Instead of sexual dimorphism, a few temperate species exhibit distinct seasonal dimorphism, with puparia of summer generation(s) and overwintering puparia being markedly different (Martin et al. 2000). All the species of *Aleurochiton* Tullgren, 1907 and the European species *Pealius quercus* (Signoret, 1868) overwinter as especially robust puparia that fall to the ground on dead leaves and then yield adult whiteflies in the spring. It is probable that some other *Pealius* Quaintance & Baker, 1914 species also do this, but the biology of most whiteflies remains unknown (Wang et al. 2016). In this work, we found that this seasonal dimorphism also appeared in *Aleuroclava*. In the autumn, we were lucky to witness the process of *Aleuroclava montanus* (Takahashi, 1939) transitioning from the summer generation to the overwintering puparia. Like the species mentioned above, the overwintering puparia of *A. montanus* also secrete a thick wax coating, which is absent in summer forms. This is the first description of seasonal dimorphism in *Aleuroclava* and indicates that more whitefly species may need to be examined. For example, the specimens identified as *Aleuroclava meliosmae* (Takahashi, 1932) in the Natural History Museum in London (NHMUK) are actually the summer puparia of *A. montanus*. The difference between the two species is detailed in the Remarks of *A. montanus* below.

## Material and methods

Puparia of the species were collected from Zhejiang and Yunnan Provinces, China. The puparia were mounted following the method suggested by Martin (1987). The terminology for morphological structures follows Bink-Moenen (1983), Martin (1985), and Gill (1990). The habitus images were taken using a Nikon D500 digital camera with a Laowa 100 mm F2.8 macro 2× lens. Puparial measurements and microphotographs were taken using an Olympus (CX33) from Zhejiang Agriculture and Forestry University, Lin’an, China (**ZAFU**). These images were then stacked using Helicon Focus 8.1.0 (Helicon Soft Ltd, Kharkiv, Ukraine). The scanning electron microscope images were taken with a GeminiSEM 360 Scanning Electron Microscope (ZEISS, Germany). Adobe Photoshop (CC 2019) software was used to make small adjustments and to assemble the plates. The holotype is deposited in the Insect Collection of ZAFU.

## Taxonomy

### Genus Aleuroclava

Taxon classificationAnimaliaHemipteraAleyrodidae

Singh

349B2842-7B31-575F-91FE-A6EA2F81472A

#### Type species.

*Aleuroclava complex* Singh, 1931: 90.

#### Diagnosis.

Puparia small in size, elliptical or subelliptic, pale yellow to white or black. Margin with one row of teeth. Many species have the submargin separated from the rest of the dorsum by a thin, concentric suture just inside the lateral margin that is interrupted at the caudal furrow, dorsum generally with tubercles. Tracheal opening in the shape of a cleft (shaped like a shallow bowl with a wide base) with smooth inner margins or undifferentiated. Vasiform orifice cordate, subcordate or subcircular with posterior margin closed, notched medially in most species; operculum cordate, nearly filling orifice; caudal furrow distinct (Wang and Du 2016; Josephrajkumar et al. 2024).

### Aleuroclava bannanensis

Taxon classificationAnimaliaHemipteraAleyrodidae

Lin & Wang sp. nov.

2289FECC-2DD7-5CEA-8FF2-BE9E9AD3C05A

https://zoobank.org/14FA1D83-127C-4AAB-8245-1ACE2D09B7DD

Figs 1, 2, 3

#### Type material.

***Holotype* puparium**: China • Yunnan, Xishuangbanna Tropical Botanical Garden, 21°55'36"N, 101°15'19"E, alt. 558 m, 1 puparium on slide, 29. vii. 2023, leg. Qing-Song Lin, Lin-Qian Lu & Xiao Zhang, on unidentified plant, deposited in ZAFU. ***Paratypes***: • 3 puparia on 2 slides with same collection data as the holotype.

#### Description.

**Figure 1.**
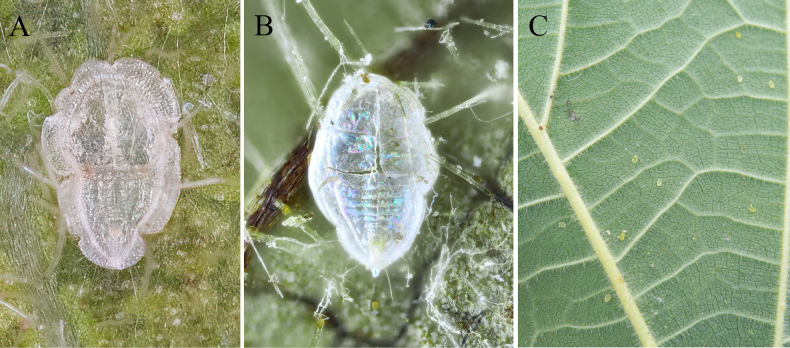
Habitus. **A**. *Aleuroclava bannanensis* sp. nov.; **B, C**. *Aleuroclava rubi* sp. nov.

***Puparium***. Empty pupal case white, elliptical in shape, with some deep indentations on the margin, slightly constricted at the thoracic and caudal ends (Figs 1A, 3A), 0.608–0.649 mm long, 0.421–0.462 mm wide. There is no obvious wax secretion on the dorsal surface and margin (Fig. 1A).

**Figure 2.**
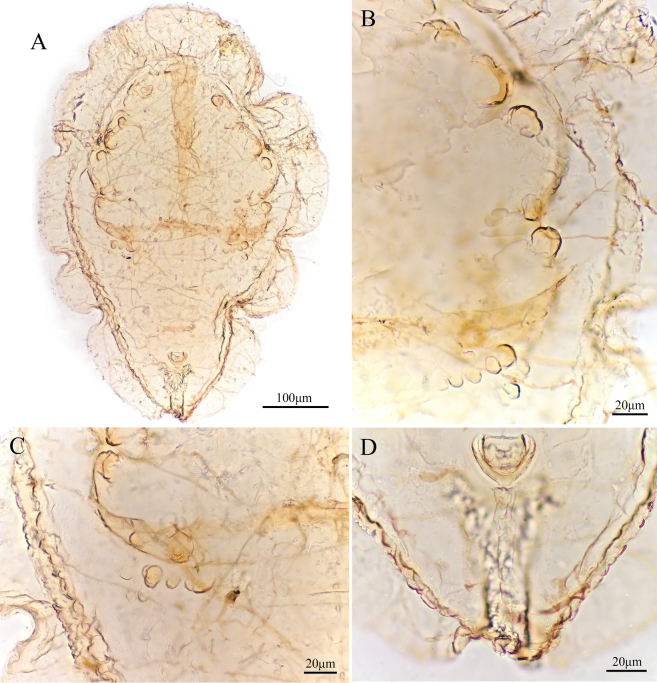
*Aleuroclava bannanensis* sp. nov., slide-mounted specimen. **A**. Puparium; **B**. Dorsum tubercles and thoracic tracheal opening; **C**. First abdominal setae and tubercles; **D**. Vasiform orifice and caudal furrow.

**Figure 3.**
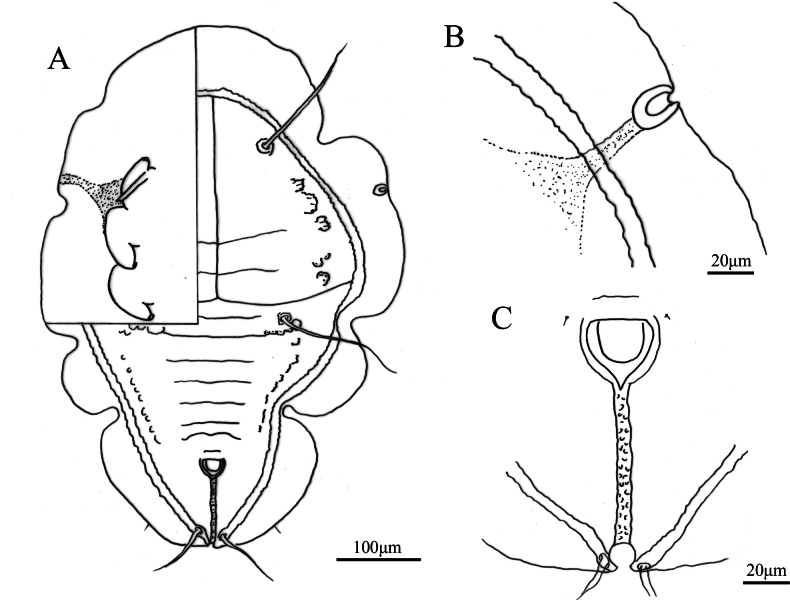
*Aleuroclava bannanensis* sp. nov., line drawings. **A**. Puparium (dorsal and ventral views); **B**. Margin and thoracic tracheal opening; **C**. Vasiform orifice and caudal furrow.

***Margin***. Faintly crenulate. Thoracic tracheal pores C-shape, inner margin smooth. Caudal furrow narrow, with some minute tubercles on it (Figs 2B, 3B).

***Dorsum***. Submargin separated from dorsal disc by a ridge. Longitudinal and transverse molting sutures both reaching submargin (Fig. 3A). The cephalic and first abdominal segments are long and situated on large tubercles (Fig. 2C). Cephalothorax with six pairs of short, blunt tubercles, and some smaller tubercles located longitudinally along the termination of the abdominal segment. Median length of pro-, meso- and metathorax: 47.8, 49.9 and 59.5 µm, respectively. Length of abdominal segments as measured along the midline as follows: abdominal segments I–V each 26.6 µm; abdominal segments VI 21.7 µm; abdominal segment VII 20.3 µm; and abdominal segment VIII 18.4 µm. Abdominal segment sutures reaching subdorsal area. Distance between posterior end of vasiform orifice and caudal opening measured 85.9 µm long (Figs 2D, 3C).

***Vasiform orifice***. Subcordate, posteriorly protrude (Figs 2D, 3C), 28.1 µm long, 33.9 µm wide; operculum subcordate, 16.7 µm long, 23.8 µm wide, almost covering the orifice; lingula concealed.

***Venter***. Thoracic and caudal tracheal folds distinct (Figs 2D, 3B). Antennae 65.7 µm long, extend through inside prothoracic legs. Legs not elevated from the ventral surface, apical pads present; adhesive sacs and spiracles visible.

***Chaetotaxy***. Posterior marginal setae 17.6 µm long. Cephalic setae 168.8 µm long. First abdominal setae 152.7 µm long. Eighth abdominal setae lateral to vasiform orifice, 3.5 µm long. Caudal setae 92.1 µm long.

#### Host plant.

Unidentified plant.

#### Distribution.

China (Yunnan).

#### Biology.

Specimens were found on the undersurface of leaves. No adults were collected in the samples, and no ants were observed attending the whitefly.

#### Etymology.

The species takes its name from its type locality, Xishuangbanna, China.

#### Remarks.

The new species differs from its congeners in having some tubercles arranged longitudinally at the termination of the abdominal segment, and the in having the submargin separated from the dorsal disc by a ridge. The puparium of the new species differs from *A. canangae* in lacking two-jointed tuberculate setae (present in *A. canangae*) and in having a cephalothorax with six pairs of short, blunt tubercles (three pairs in *A. canangae*).

### Aleuroclava rubi

Taxon classificationAnimaliaHemipteraAleyrodidae

Lin & Wang sp. nov.

3030CF98-196E-5375-ABF3-A52EC11CDC63

https://zoobank.org/96959AAC-58A3-45D7-B65D-DF573933D781

Figs 1, 4–6

#### Type material.

***Holotype* puparium**: China, • Zhejiang, Hangzhou, Thousand Island Lake, 29°41'56"N, 118°55'18"E, alt. 428 m, 1 puparium on slide, 12. vi. 2024, leg. Qing-Song Lin, on *Rubus lambertianus*, deposited in ZAFU. ***Paratypes***: 13 puparia on 4 slides with same collection data as the holotype.

#### Description.

***Puparium***. Yellow in life, empty pupal case white, oval shaped, slightly narrowed posteriorly (Fig. 1B, 1C), 0.554–0.586 mm long, 0.344–0.368 mm wide. There is no obvious wax secretion on the dorsal surface and margin.

**Figure 4.**
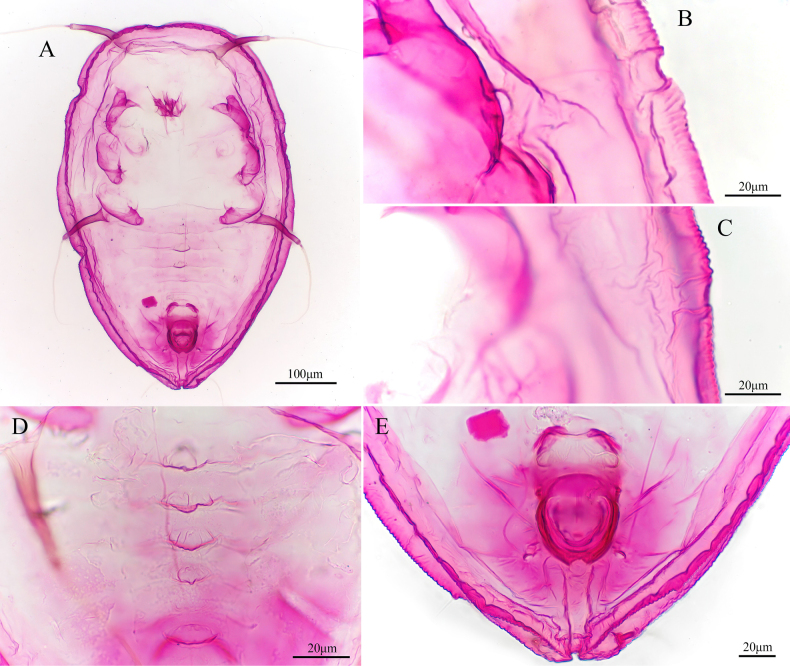
*Aleuroclava rubi* sp. nov., slide-mounted specimen. **A**. Puparium; **B, C**. Margin and thoracic tracheal opening; **D**. Median abdominal tubercles; **E**. Vasiform orifice and caudal furrow.

**Figure 5.**
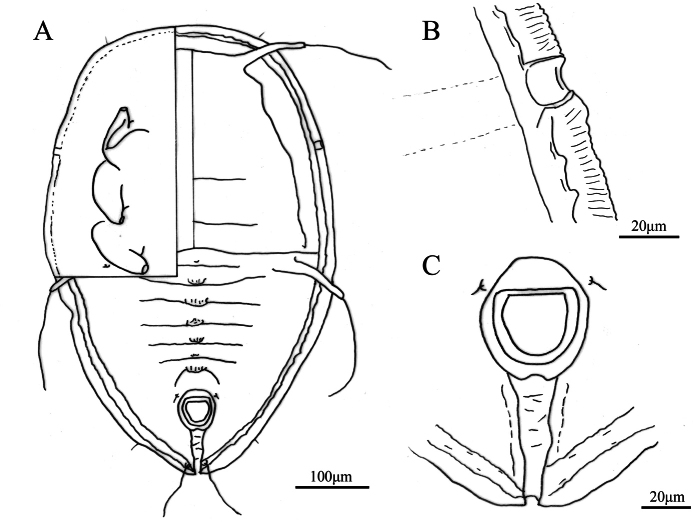
*Aleuroclava rubi* sp. nov., line drawings. **A**. Puparium (dorsal and ventral views); **B**. Margin and thoracic tracheal opening; **C**. Vasiform orifice and caudal furrow.

***Margin***. Faintly crenulate, with 11–14 crenulations in 0.1 mm. Thoracic tracheal opening in the shape of a cleft (shaped like a shallow bowl with a wide base) with smooth inner margins, not pore-like (Figs 4C, 5B). Caudal furrow without tuberculate, but with some indistinct transverse folds on it (Figs 4E, 5C).

***Dorsum***. Submargin faintly separated from dorsal by a fold. Longitudinal and transverse molting suture both reaching submargin (Fig. 5A). Cephalic and first abdominal segment setae extremely long and tuberculate and set on tubercles. Abdominal segments I to VII with median tubercle, small or absent in I/VI (Figs 4D, 5A). Median length of pro-, meso- and metathorax: 90.8, 56.6 and 47.1 µm, respectively. Length of abdominal segments as measured along the midline as follows: abdominal segments I–V each 23.2 µm; abdominal segment VI 18.9 µm; abdominal segments VII–VIII each 16.3 µm. Distance between posterior end of vasiform orifice and caudal opening measured 60.4 µm long.

**Figure 6.**
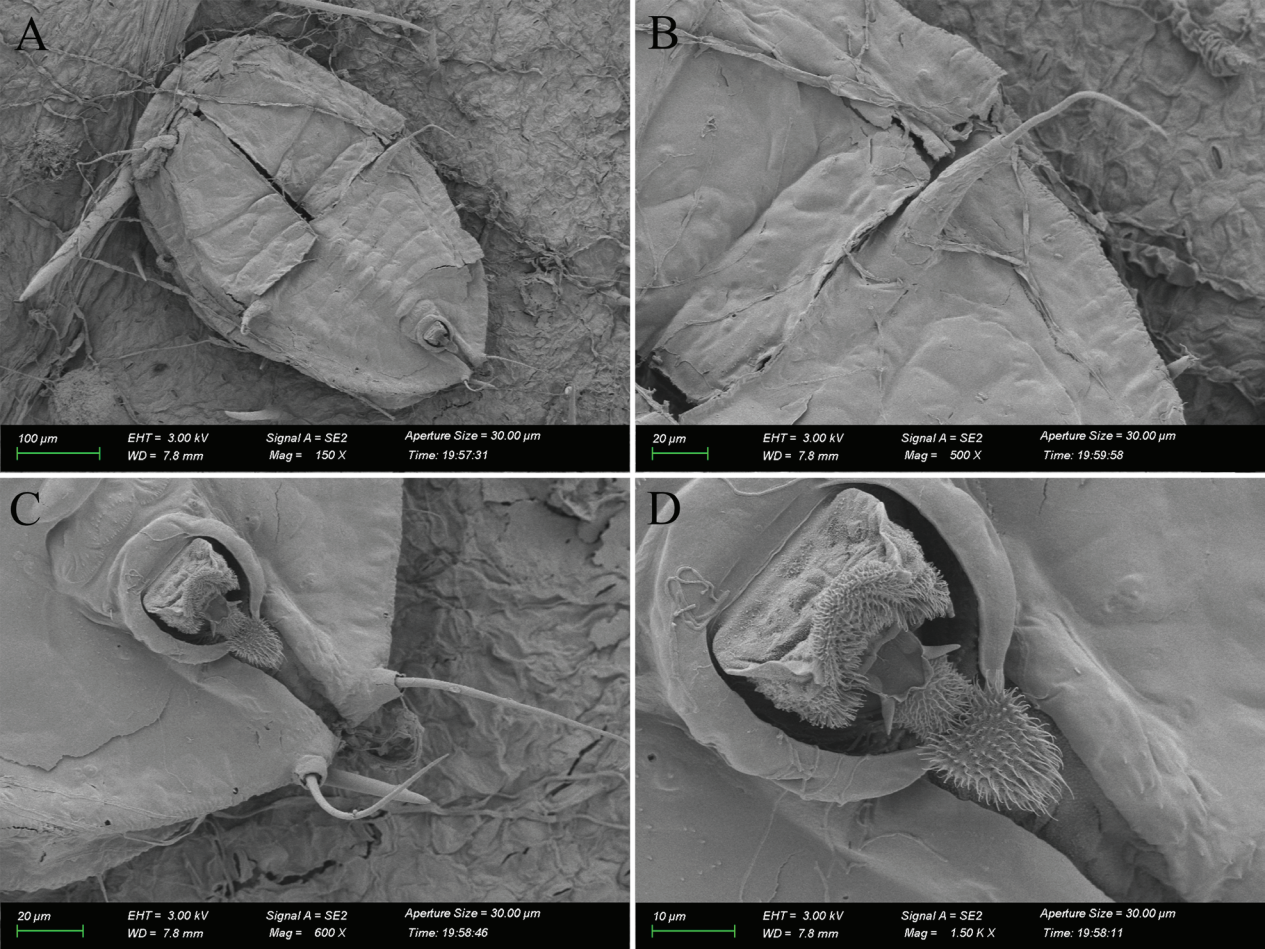
*Aleuroclava rubi* sp. nov., scanning electron microphotographs. **A**. Puparium; **B**. First abdominal setae; **C**. Vasiform orifice and caudal tracheal furrow; **D**. Vasiform orifice.

***Vasiform orifice***. Cordate, with a cleft along the posterior margin medially (Fig. 4E), 52.2 µm long, 48.1 µm wide; operculum subcordate, 29.0 µm long, 27.6 µm wide, almost covering the orifice; lingula concealed and with setae (Figs 4E, 6D).

***Venter***. The narrow submarginal area is faintly indicated on the ventral margin. Thoracic and caudal tracheal folds indistinct, thoracic tracheal cleft present. Antennae 48.1 µm long. Anterior abdominal spiracle present.

***Chaetotaxy***. Anterior marginal setae 21.6 µm long. Posterior marginal setae 25.1 µm long Cephalic setae 392.2 µm long (apical joint 277.8 µm and basal joint 114.4 µm). First abdominal setae 288.3 µm long (apical joint 186.2 µm and basal joint 102.1 µm). Eighth abdominal setae lateral to vasiform orifice, 4.5 µm long. Caudal setae 120.2 µm long.

#### Host plant.

*Rubus lambertianus* (Rosales, Rosaceae).

#### Distribution.

China (Zhejiang).

#### Biology.

Specimens were found with approximately 10–20 puparia per leaf on the undersurface. No ants were observed attending the whitefly.

#### Etymology.

The species is named for *Rubus*, the genus of its host plant.

#### Remarks.

The puparium of the new species closely resembles that of *Aleuroclava srilankaensis* (David, 1993), but differs by having a median tubercle on abdominal segments II–V and VII (vs. II–IV in *A. srilankaensis*), and a distinct thoracic tracheal opening (absent in *A. srilankaensis*). Type specimens of *A. srilankaensis* were not available to the authors for examination, and has it also not been recorded in China.

### Aleuroclava yunnanensis

Taxon classificationAnimaliaHemipteraAleyrodidae

Lin & Wang sp. nov.

AA75EF8B-DE39-57E9-9EEF-84EA31EAB03D

https://zoobank.org/ADDA2818-C1DE-4DDF-B10F-9E5FEDDFC47C

Figs 7, 8

#### Type material.

***Holotype* puparium**: China • Yunnan, Xishuangbanna Tropical Botanical Garden, 21°55'36"N, 101°15'19"E, alt. 558 m, 1 puparium on slide, 29. vii. 2023, leg. Qing-Song Lin, Lin-Qian Lu & Xiao Zhang, on *Baccaurea ramiflora*, deposited in ZAFU.

#### Description.

**Figure 7.**
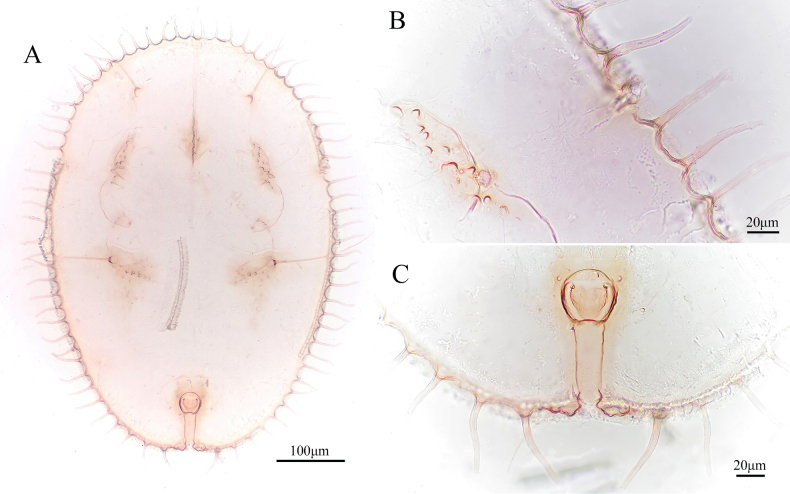
*Aleuroclava yunnanensis* sp. nov., slide-mounted specimen. **A**. Puparium; **B**. Margin and thoracic tracheal opening; **C**. Vasiform orifice and caudal furrow.

***Puparium***. Empty pupal case white with some dark pigments, elliptical in shape, broadest at the transverse moulting suture region, 0.649 mm long, 0.451 mm wide, without secretion of wax (Fig. 7A).

***Margin***. Faintly crenulate. Thoracic tracheal folds and pores distinct. Caudal furrow is as wide as the operculum, without tuberculate on it (Fig. 7B, C).

**Figure 8.**
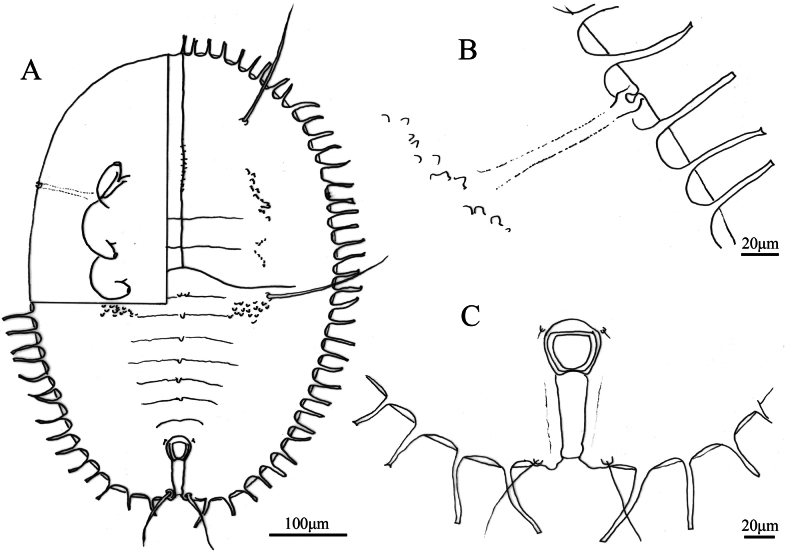
*Aleuroclava yunnanensis* sp. nov., line drawings. **A**. Puparium (dorsal and ventral views); **B**. Margin and thoracic tracheal opening; **C**. Vasiform orifice and caudal furrow.

***Dorsum***. Group of small tubercles located at the termination of the prothorax and the second abdominal segment. Submargin with a series of long papillae, 36 pairs, reaching beyond margin, 56.6 µm long. Longitudinal molting suture reaching margin and transverse molting suture reaching subdorsal area (Fig. 8A). Abdominal segment I–VI with a median tubercle. Median length of pro-, meso- and metathorax: 45.8, 52.7 and 37.6 µm, respectively. Length of abdominal segments as measured along the midline as follows: abdominal segment I 40.2 µm; abdominal segments II–V each 32.6 µm; abdominal segment VI 28.9 µm; abdominal segments VII–VIII each 25.8 µm. Distance between posterior end of vasiform orifice and caudal opening measured 61.8 µm long.

***Vasiform orifice***. Subcordate, posterior notched (Figs 7C, 8C), 35.0 µm long, 37.4 µm wide; operculum subcordate, 20.8 µm long, 24.5 µm wide, almost covering the orifice; lingula concealed.

***Venter***. Thoracic tracheal fold narrow and caudal tracheal fold wide, without stipples. Antennae 64.7 µm long, extend through inside prothoracic legs.

***Chaetotaxy***. First abdominal setae 294.5 µm long. Eighth abdominal setae lateral to vasiform orifice, 5.2 µm long. Caudal setae 122.6 µm long.

#### Host plant.

*Baccaurea ramiflora* (Malpighiales, Phyllanthaceae).

#### Distribution.

China (Yunnan).

#### Biology.

Specimens were found singly on the undersurface of leaves. No adults were collected in the samples, and no ants were observed attending the whitefly.

#### Etymology.

The species takes its name from its type locality, Yunnan Province, China.

#### Remarks.

The puparium of the new species closely resembles that of *Aleuroclava filamentosa* (Corbett, 1933), but differs in having several groups of small tubercles with dark pigments (absent in *A. filamentosa*). In addition, through examination of the paratype of *A. filamentosa* at NHMUK, it was found that the presence of a pair of setae on each abdominal segment in *A. filamentosa* should be a characteristic of its endoparasitic wasp (this feature was not observed in non-parasitized specimens) (Fig. 13). The distinguishing features mentioned in the new species described by Dubey (2020b) are actually present in *A. filamentosa* as well (such as the median tubercles on the abdomen), but a synonym is not proposed here.

##### New record for China

### Aleuroclava bifurcata

Taxon classificationAnimaliaHemipteraAleyrodidae

(Corbett, 1933)

637C316F-3A4D-5C0B-B57B-A9E2F44F5839

Figs 9, 10

Dialeurodes bifurcata Corbett, 1933: 126–127.
Aleurotuberculatus bifurcata (Corbett); Mound and Halsey 1978: 80.
Aleuroclava bifurcata (Corbett); Jesudasan and David 1990: 3; Martin 1999: 31; Martin and Mound 2007: 10.

#### Host plant.

Sapindaceae: *Nephelium mutabile*, *Nephelium lappaceum* (Evans, 2008); Phyllanthaceae: *Baccaurea ramiflora*.

#### Distribution.

China (Yunnan); Malaysia (Evans 2008).

#### Material examined.

China • Yunnan, Wangtianshu Scenic Spot, 21°37'18"N, 101°35'17"E, alt. 755 m, 4 puparia on 1 slide, 1. viii. 2023, leg. Qing-Song Lin, Lin-Qian Lu & Xiao Zhang, on *Baccaurea ramiflora*, deposited in ZAFU.

**Figure 9.**
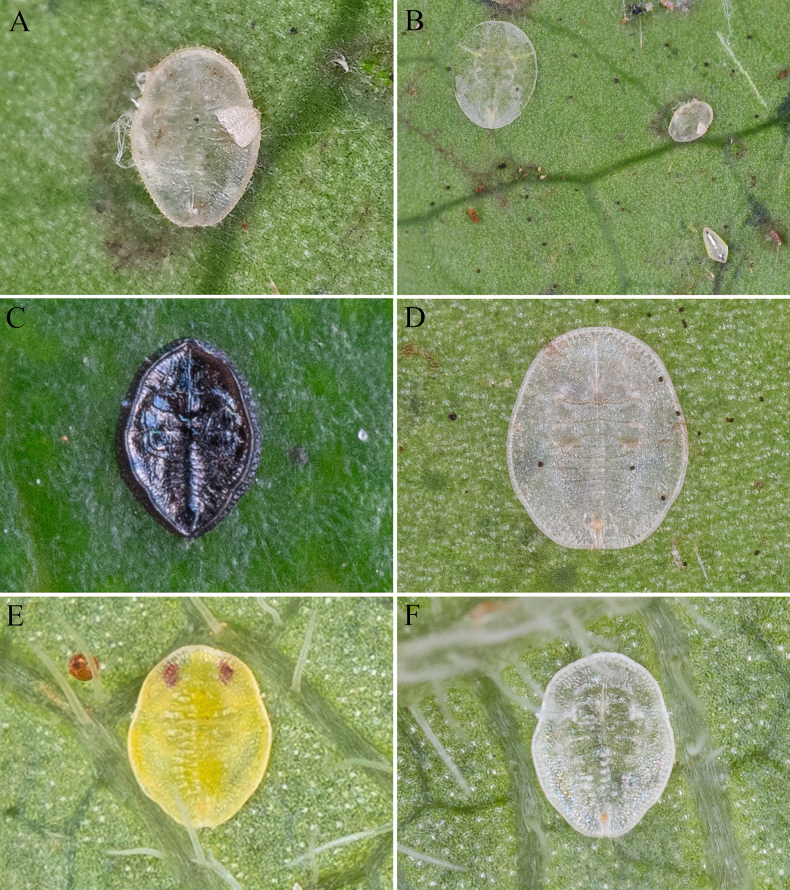
Four new record species from China, habitus. **A**. *Aleuroclava bifurcata*; **B**. *A. bifurcata* (upper right); **C**. *A. citrifolii*; **D**. *A. pearlis*; **E, F**. *A. stereospermi*.

**Figure 10.**
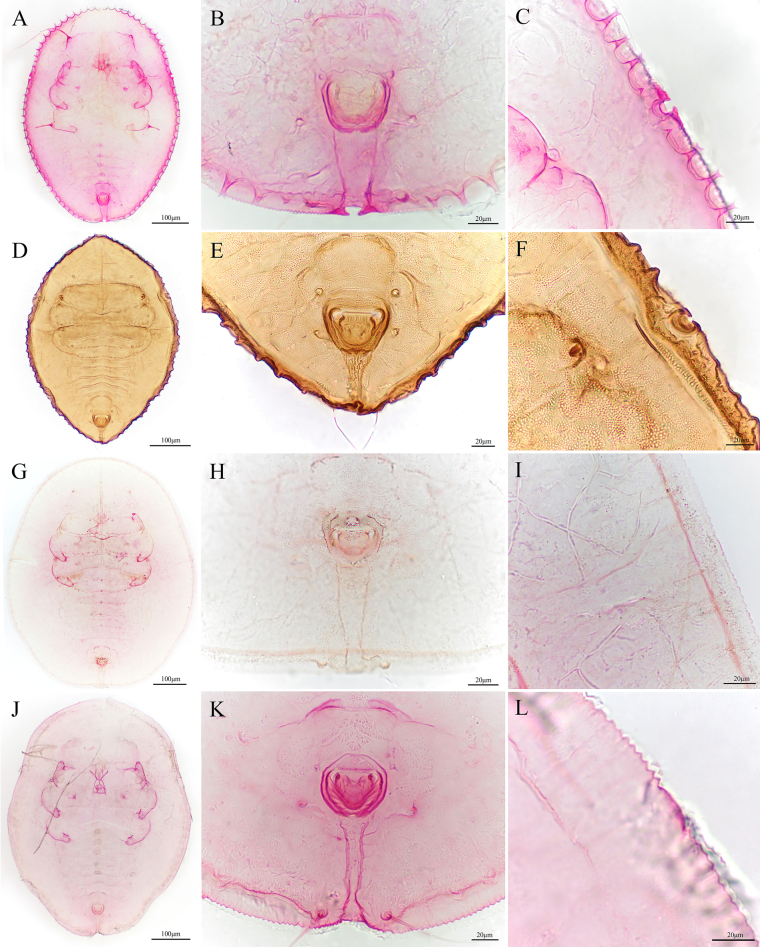
Four new record species from China, slide-mounted specimens: *Aleuroclava bifurcata*. **A**. Puparium; **B**. Vasiform orifice; **C**. Margin; *A. citrifolii*; **D**. Puparium; **E**. Vasiform orifice; **F**. Margin; *A. pearlis*; **G**. Puparium; **H**. Vasiform orifice; **I**. Margin; *A. stereospermi*; **J**. Puparium; **K**. Vasiform orifice; **L**. Margin.

#### Remarks.

The examined specimens agree with *Aleuroclava bifurcata* in having cephalic and first abdominal setae that extend beyond the body margin, median tubercles on the abdominal segments, and a matching number of marginal setae. The only divergence lies in the thoracic and caudal tracheal openings, which are distinctly thickened and sharpened in our specimens.

### Aleuroclava citrifolii

Taxon classificationAnimaliaHemipteraAleyrodidae

(Corbett, 1935)

1AA056D9-3CE6-51A1-827E-C3F5FCC10636

Figs 9, 10

Aleurolobus citrifolii Corbett, 1935: 9.
Aleurotuberculatus citrifolii (Corbett); Mound and Halsey 1978: 81.
Aleuroclava citrifolii (Corbett); Martin 1999: 31; Martin and Mound 2007: 10.

#### Host plant.

Rutaceae: *Citrus* sp., *Murraya exotica* (Evans 2008); Juglandaceae: *Juglans regia*.

#### Distribution.

China (Yunnan); India, Pakistan (Evans 2008).

#### Material examined.

China, • Yunnan, Xishuangbanna Tropical Botanical Garden, 21°55'26"N, 101°15'14"E, alt. 562 m, 3 puparia on 2 slides, 28. vii. 2023, leg. Qing-Song Lin, Lin-Qian Lu & Xiao Zhang, on *Juglans regia*, deposited at ZAFU.

#### Remarks.

This species can be distinguished from other *Aleuroclava* species by the following combination of characteristics: large thoracic tracheal openings, a small pointed tubercle in the middle of the posterior vasiform orifice, and a caudal furrow with irregularly shaped linear markings.

### Aleuroclava pearlis

Taxon classificationAnimaliaHemipteraAleyrodidae

(Pushpa & Sundararaj, 2010)

BB27E36A-095E-55E3-8C7E-296897296FB0

Figs 9, 10

Minutaleyrodes pearlis Pushpa & Sundararaj, 2010: 47–48.
Aleuroclava pearlis (Pushpa & Sundararaj); Dubey 2020a: 327.

#### Host plant.

Myrtaceae: *Syzygium* sp. (Pushpa and Sundararaj 2010), *Syzygium malaccense*.

#### Distribution.

China (Hong Kong, Yunnan); India (Pushpa and Sundararaj 2010).

#### Material examined.

China • Yunnan, Xishuangbanna Tropical Botanical Garden, 21°53'26"N, 101°16'14"E, alt. 534 m, 2 puparia on 2 slides, 29. vii. 2023, leg. Qing-Song Lin, Lin-Qian Lu & Xiao Zhang, on *Syzygium malaccense*, deposited in ZAFU. China, Hong Kong, 5 puparia on 1 slide, 26. x. 1990, leg. JH Martin, on *Syzygium hancei*, deposited in NHMUK.

#### Remarks.

After examining the specimens at NHMUK, the “*Aleuroclava*, undetermined sp. 2”, which has not been identified in Hong Kong (Martin and Lau 2011), also belongs to this species.

### Aleuroclava stereospermi

Taxon classificationAnimaliaHemipteraAleyrodidae

(Corbett, 1935)

B6401361-EA5A-54BE-92FA-D39576A91918

Figs 9, 10

Aleurotuberculatus stereospermi Corbett, 1935: 832.
Aleuroclava stereospermi (Corbett); Jesudasan and David 1990: 4; Martin 1999: 31; Martin and Mound 2007: 12.

#### Host plant.

Bignoniaceae: *Stereospermum chelonoides* (Evans 2008); Vitaceae: *Leea indica*.

#### Distribution.

China (Yunnan); Malaysia (Evans 2008).

#### Material examined.

China • Yunnan, Pu’er, Xiaohei River Forest Park, 23°12'32"N, 100°55'54"E, alt. 951 m, 7 puparia on 7 slides, 29. vii. 2023, leg. Qing-Song Lin, Lin-Qian Lu & Xiao Zhang, on *Leea indica*, deposited in ZAFU.

#### Remarks.

This species can be distinguished from other *Aleuroclava* species by the following combination of characteristics: tracheal openings indicated by the absence of crenulations and terminating in a shallow concavity, operculum narrowing posteriorly.

### Aleuroclava montanus

Taxon classificationAnimaliaHemipteraAleyrodidae

(Takahashi, 1939)

0EC921B5-FA2A-59C4-8E28-95108B1FD773

Figs 11, 12

Taiwanaleyrodes montanus Takahashi, 1939: 80.
Aleuroclava montanus (Takahashi); Manzari and Quicke 2006: 2470; Martin and Mound 2007: 11.

#### Host plant.

Daphniphyllaceae: *Daphniphyllum teijsmanni* (Suh 2009); Lauraceae (Takahashi 1939), *Camphora officinarum*, *Sassafras tzumu*.

#### Distribution.

China (Yunnan, Zhejiang, Taiwan [Takahashi 1939]); Korea (Suh 2009).

#### Material examined.

China • Zhejiang, Hangzhou, 30°07'40"N, 120°01'33"E, alt. 98 m, 15 puparia on 4 slides, 21. xi. 2024, leg. Jian-Bo Li, on *Sassafras tzumu*; China, • Zhejiang, Xinchang, 15 puparia on 4 slides, 14. viii. 2024, leg. Qing-Song Lin, on *Sassafras tzumu*; China, • Yunnan, Kunming, Kunming Botanical Garden, 2 puparia on slide, 15. x. 2016, leg. JR Wang, on *Michelia cavaleriei*; China, • Taiwan, 1 puparium on slide, 17. vii. 1994, leg. C. C. Ko, on *Camphora officinarum*. All deposited in ZAFU.

#### Remarks.

**Figure 11.**
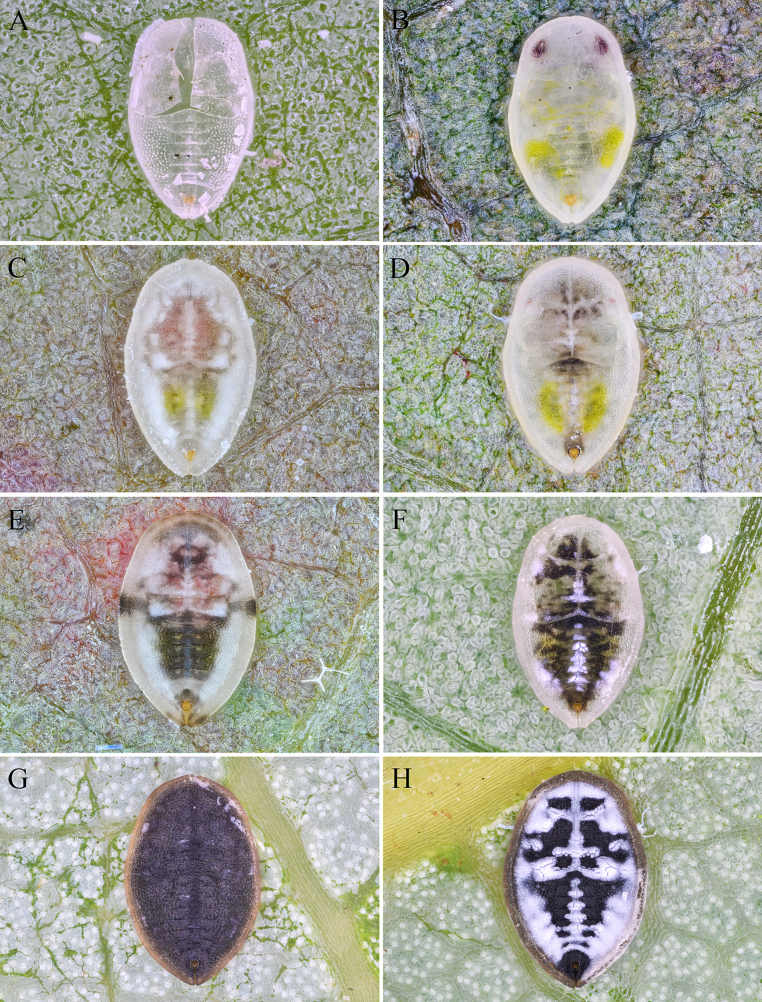
*Aleuroclava montanus*, habitus. **A–H**. Transformation process from summer generation to overwintering puparia.

**Figure 12.**
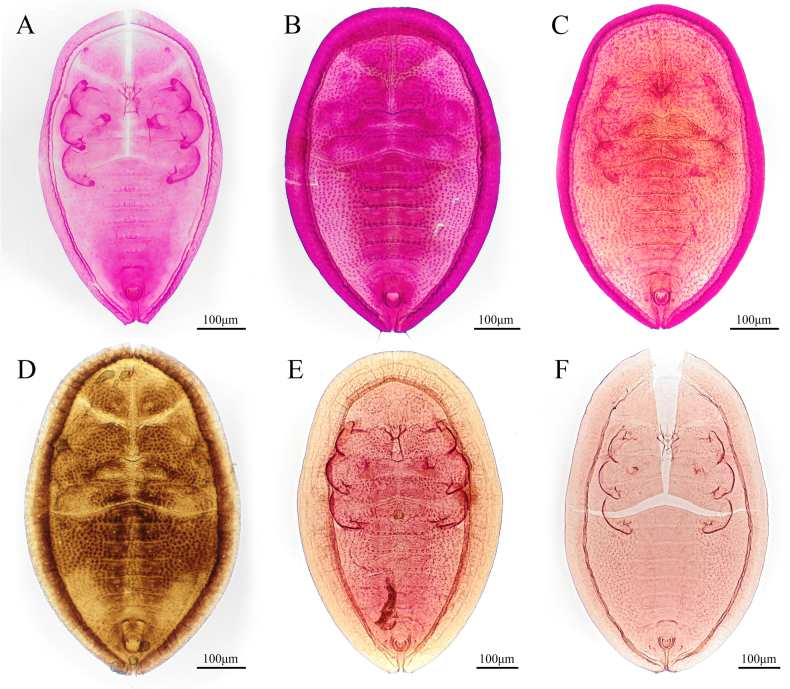
Puparia, slide-mounted specimens. **A**. *Aleuroclava montanus* summer puparia; **B, C**. *A. montanus* intermediate type; **D**. *A. montanus* overwintering puparia; **E**. *A. montanus* from Taiwan; **F**. *A. meliosmae*.

This is the first discovery of the seasonal dimorphism in *Aleuroclava*. Summer puparia have a pale cuticle (Fig. 11A, B), whereas mature overwintering puparia are dark, heavily sclerotized and secrete a dense coating of white wax on the dorsal surface (Fig. 11H). We found only pale puparia on the same host during the summer of the same year, with no definitive identification, until seasonal dimorphism was discovered in the autumn. The summer form of *A. montanus* is easily confused with *A. meliosmae*, and specimens identified as *A. meliosmae* at NHMUK are actually *A. montanus*. *Aleuroclava meliosmae* lacks transverse tubercles along the abdomen segments, and its caudal furrow is narrower (Fig. 12F). In addition, there is variation in the width of the ventral marginal fold in *A. montanus*, with our specimens narrower than those from Taiwan (Fig. 12E). But specimens from Korea that look like ours (Suh 2009) were identified as *A. montanus* by C. C. Ko.

**Figure 13.**
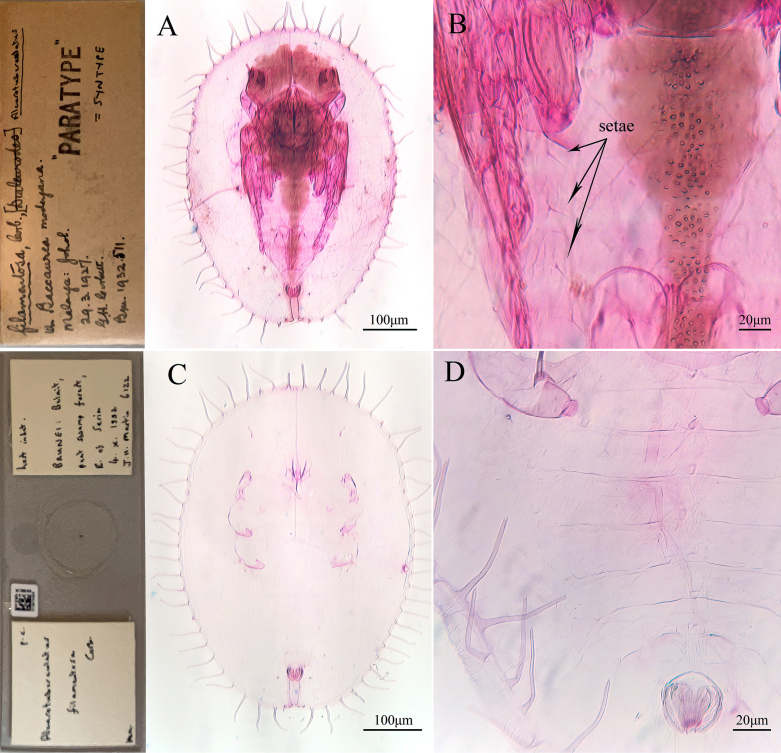
Paratype of *Aleuroclava filamentosa*. **A**. Parasitized specimen; **B**. Abdominal segment showing setal arrangement; **C**. Non-parasitized specimen; **D**. Abdominal segment lacking paired setae.

## Supplementary Material

XML Treatment for Aleuroclava

XML Treatment for Aleuroclava bannanensis

XML Treatment for Aleuroclava rubi

XML Treatment for Aleuroclava yunnanensis

XML Treatment for Aleuroclava bifurcata

XML Treatment for Aleuroclava citrifolii

XML Treatment for Aleuroclava pearlis

XML Treatment for Aleuroclava stereospermi

XML Treatment for Aleuroclava montanus

## Additional information

### Use of AI

No use of AI was reported.

### Author ORCIDs

Qing-Song Lin https://orcid.org/0000-0002-7757-1907

Ji-Rui Wang https://orcid.org/0000-0002-8388-1565

### Data availability

All of the data that support the findings of this study are available in the main text.
